# Glycoprotein non-metastatic melanoma protein B is a biomarker of inflammation in individuals with Gaucher disease: relationship to clinico-pathological subtypes

**DOI:** 10.1186/s13023-025-04054-y

**Published:** 2025-10-28

**Authors:** Sebile Kilavuz, Kerri-Lee Wallom, Ana Catarina Gomes Almeida Augusto Caçote, Aimée Donald, Simona D’Amore, Kathy Page, Chong Yew Tan, Danielle te Vruchte, Andrea Sturchio, Panagiota Tsitsi, Ellen Hertz, Mattias Andréasson, Ioanna Markaki, Per Svenningsson, Timothy M. Cox, Frances M. Platt, the MRC GAUCHERITE Consortium

**Affiliations:** 1 Department of Pharmacology, University of Oxford, Mansfield Road, Oxford, OX1 3QT UK; 2 Division of Pediatric Metabolism and Nutrition, Department of Pediatrics, Marmara University Faculty of Medicine, Istanbul, Turkey; 3 Division of Neurosciences, Faculty of Biology, Medicine & Health, University of Manchester, Manchester, UK; 4 Department of Medicine, Cambridge Lysosomal Storage Disorders Unit, Royal Free Hospital NHS Foundation Trust, University of Cambridge, London, UK; 5 Department of Precision and Regenerative Medicine - Ionian Pole, University of Bari Aldo Moro, Bari, Italy; 6 Department of Medicine, University of Cambridge, Cambridge, UK; 7 Department of Clinical Neuroscience, Karolinska Institute, Stockholm, Sweden

**Keywords:** Gaucher disease, gpNMB, Liver disease, Monoclonal gammopathy, Pulmonary disease, Parkinson’s disease, Inflammation

## Abstract

**Background:**

Gaucher disease (GD) is a lysosomal disease caused by mutations in the *GBA1* gene, leading to glucosylceramide and glucosylsphingosine accumulation. *GBA1* mutations are also the most common genetic risk factor for Parkinson’s disease (PD). Increased expression of glycoprotein non-metastatic melanoma protein B (gpNMB), a potential biomarker of inflammation and neurodegeneration, has been reported in PD, GD and other LSDs. Plasma concentrations of gpNMB are correlated with the accumulation of bioactive lipid substrates in several chronic inflammatory diseases and gpNMB stimulates lipogenesis in white adipocytes. To explore its potential significance in GD we measured plasma gpNMB in patients with Gaucher Disease type 1 (GD1), Gaucher Disease Type 3 (GD3), GD1-PD, PD and *GBA* heterozygous PD and in different clinicopathological subtypes.

**Results:**

The study enrolled participants the GAUCHERITE Cohort in the UK (172 GD1 and 20 GD3 patients) and the Biopark Cohort (72 IPD patients) in Sweden. Plasma concentrations of gpNMB were significantly higher in patients with Gaucher disease (mean: 200.9; range: 9.8-1643 ng/ml) compared with healthy controls (mean: 35.1; range.: 10.1- 125 ng/ml), including those receiving enzyme replacement therapy (ERT). Notably, gpNMB concentrations remained elevated in GD1 patients who had received ERT for more than 5 years. The biomarker was particularly elevated in patients who had been splenectomized, those with known pulmonary or liver disease, and those with monoclonal gammopathy, despite enzyme therapy. No statistical difference was found in plasma gpNMB concentrations between treated patients with GD1 and GD3. On average, there was no difference in plasma gpNMB concentrations between Gaucher patients with or without Pakinsonism. As expected however plasma gpNMB concentrations among patients with Parkinsonism were higher in those with type 1 Gaucher disease than either *GBA1* heterozygotes or those with idiopathic PD (p=0.0001).

**Conclusion:**

Our findings indicate that the association of plasma gpNMB with liver cirrhosis, gammopathy and pulmonary disease in Gaucher disease warrants further investigation. Additionally, plasma gpNMB may serve as a supportive biomarker in the evaluation and clinical monitoring of residual disease activity. However, plasma gpNMB neither differentiated between the neuronopathic subtypes of Gaucher disease nor idiopathic Parkinson’s disease.

**Supplementary Information:**

The online version contains supplementary material available at 10.1186/s13023-025-04054-y.

## Introduction

Gaucher disease (GD) is a disorder of lysosomal sphingolipid metabolism caused by a deficiency in lysosomal acid β-D-glucosylceramidase (GCase) and due principally to biallelic mutations in the *GBA1* gene (OMIM: 60,646) that maps to human chromosome 1q22 [1]. The frequency of this disease is highly variable across different populations and is highest in Ashkenazi Jews [2–4]. Estimated birth incidence in general populations ranges from 0.33 to 5.8 per 100 000 [4–12].

Deficiency of lysosomal GCase leads to accumulation of glucosylceramides (GlcCer) of different acylchain length and composition as well as the deacylated and diffusible metabolite, glucosylsphingosine (GlcSph), principally in the lysosomes and preferentially in macrophages [13–15]. Lipid-laden macrophages, eponymously termed ‘Gaucher’ cells, accumulate in the spleen, liver, bone marrow, lymph nodes, lung, the luminal surface of blood vessels and in the periadventitial space of cerebral arterioles. Although much studied, these pathological and alternatively activated macrophages and their secretory products are yet to be fully characterized [13–16].

Since 1961, GD is classified into three subtypes: non-neuronopathic type 1 and neuronopathic types 2 and 3. Non-neuronopathic type 1 Gaucher disease (GD1- OMIM- Online Mendelian Inheritance in Man 230800) is the most prevalent subtype in Western countries, characterized by highly variable clinical features that range from mildly symptomatic individuals to patients with visceral (liver and splenic enlargement and liver fibrosis and failure), haematological (anaemia, leukopenia, thrombocytopenia and coagulation abnormalities), skeletal (osteoporosis, osteopenia, bone pain, osteonecrosis, lytic lesions, bone crises and Erlenmeyer flask deformities), pulmonary manifestations (interstitial lung disease, alveolar, lobar consolidation and pulmonary arterial hypertension), hepato-pulmonary syndrome and malignancy (multiple myeloma, hepatocellular carcinoma and non-Hodgkin lymphoma) [17]. B cells polyclonal or monoclonal proliferation may result in myeloma and related B-cell cancers [18, 19]. Other types of GD are acute neuronopathic type 2 (OMIM 230,900 GD2) and chronic neuronopathic type 3 (OMIM 231,000 GD3). The former represents the rapidly progressive neurological form with onset in the neonatal, early and late-infantile periods, typically resulting in death before 2–3 years of age [20]. GD3, typically with onset in late-infancy and childhood, is characterized by horizontal saccadic eye movement abnormalities [21], ataxia, nerve deafness, variable cognitive impairment, pyramidal signs, kyphosis, and seizures including myoclonic epilepsy. There is marked phenotypic heterogeneity within GD3 but compared with the non-neuronopathic disease, the systemic manifestations are severe, for example in the lung, and progress rapidly without molecular therapy [17, 22–24]. Beyond the extensive systemic disease in macrophage-rich tissues, neuronopathic Gaucher disease is characterized by greatly increased concentrations of glucosylceramides and glucosylsphingosine in nervous tissue, particularly in cerebral grey matter [25, 26]. A very rare clinical phenotype of GD has been designated type 3c (OMIM 231005) in which mild neurological manifestations occur uniquely in patients homozygous for the D409H *GBA1* mutation: here calcification of the aortic and mitral valves, ascending aorta and occasionally coronary arteries, is accompanied by corneal opacities and low-pressure hydrocephalus [27, 28].

A minority (≈5%) of middle-aged and elderly patients with GD1 develop Parkinson’s Disease (PD) and dementia with Lewy bodies (DLB) [29–31] but the frequency is greatly increased over the healthy population control subject has also been shown that GCase activity is decreased in normal ageing and in the substantia nigra and putamen of patients with PD together with characteristic deposits of phosphylated alpha-synuclein [32, 33]. This clinical phenotype has confounded clinical classification of Gaucher disease since the repeated, albeit uncommon co-occurrence of PD and or LBD provides convincing evidence of pathological engagement of the nervous system in patients otherwise considered to have ‘non-neuronopathic’ disease [34]. While lysosomal dysfunction and sphingolipids are implicated in the development of Parkinson’s disease in patients with Gaucher disease and their first-degree relatives, no clear mechanistic pathway for *GBA1-*related Parkinson’s disease and Lewy body dementia has yet been established [35].

Macrophage-targeted recombinant human glucosylceramidase therapy given parenterally for Gaucher disease has been available since 1992 [36]. Imiglucerase, velaglucerase alfa and taliglucerase alfa are currently the approved enzyme replacement therapies (ERTs) although taliglucerase-alpha has not been approved by the European Medicines Agency [37–39]. In treating patients with Gaucher Disease, substrate reduction therapy (SRT) offers an alternative approach. Miglustat is occasionally used in individuals with mild to moderate Gaucher Disease as a secondline treatment when ERT is not a viable option [40]. Eliglustat has been shown to improve and stabilize the clinical features of the disease in untreated patients or patients switched from ERT [41, 42]. Eliglustat was approved as a first-line therapy (Cerdelga®) for Gaucher disease type 1 (by the FDA in 2014 and the EMA in 2015) for patients (the majority) genotypes predicting extensive, intermediate, or poor metabolism. It is generally not prescribed during pregnancy or breastfeeding, or in women of childbearing age who are not using contraception [43, 44].

Even though individuals with GD2 do not respond to either ERT or SRT (in practice eliglustat), the systemic manifestations of patients with GD3, even those sometimes classified as type 2/3 with severe somatic disease in infancy can be rescued by ERT and, as licensed in Japan, ERT combined with eliglustat as the SRT agent. Although systemic therapies may prolong life by ameliorating disease outside the nervous system, pulmonary infiltration as well as massive lymphadenopathy in thoracic as well as abdominal compartments is almost invariably refractory; and none of the existing drugs alters the prognosis of progressive neurological disease in any form of Gaucher disease [45, 46].

**Fig. 1:**
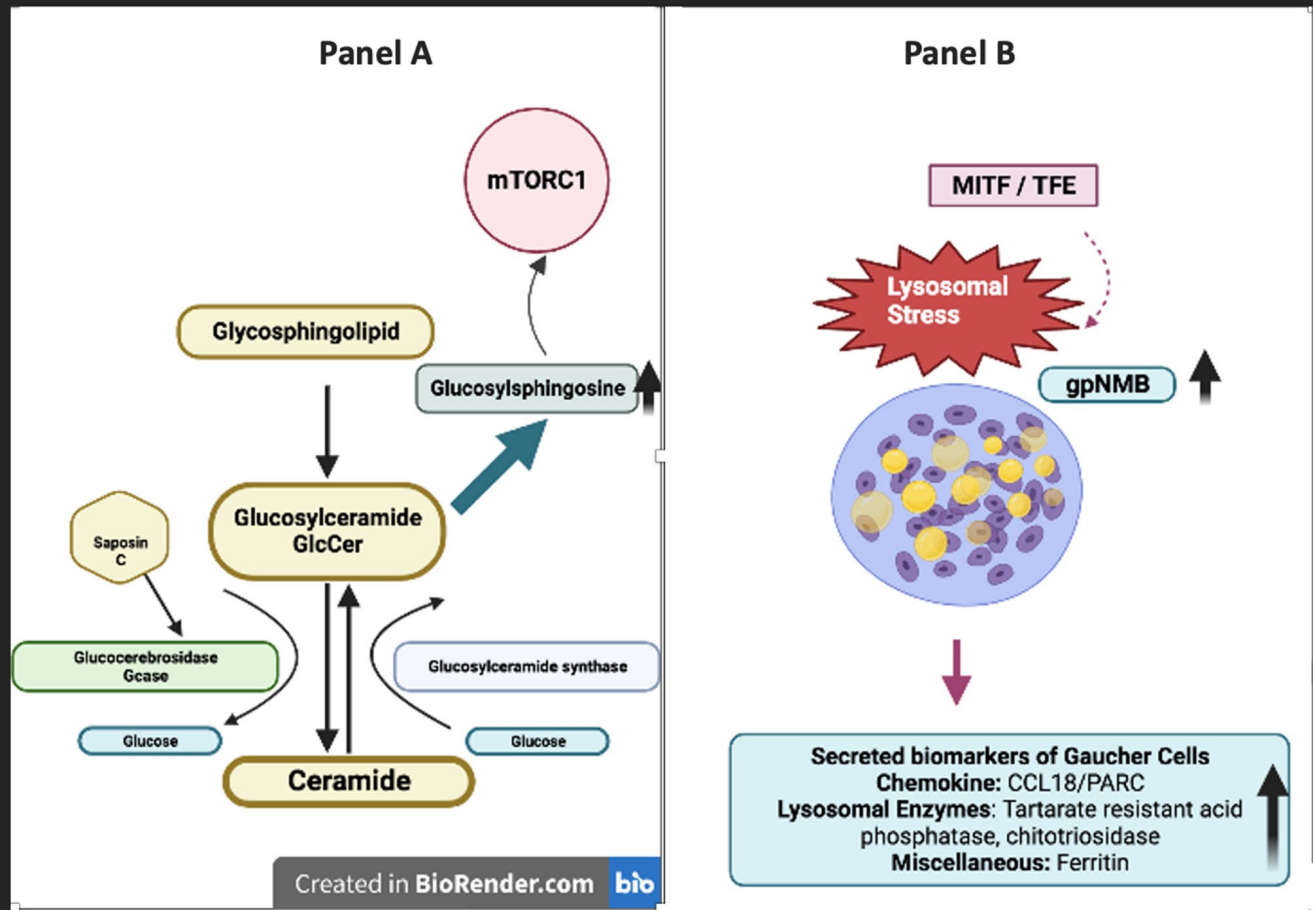
Schematic representation of Gaucher disease pathogenesis. Panel **A**: Gaucher cells accumulate the glycosphingolipid glucosylceramide (GlcCer) in the lysosome. Glucosylsphingosine is generated by the action of acid ceramidase from the GlcCer stored in the lysosome. The accumulation of GlcCer and its deacylated derivative, glucosylsphingosine, due to deficient glucocerebrosidase (GCase) activity leads to aberrant activation of mTORC1 signaling, which is implicated in impaired lysosomal homeostasis. Panel **B**: panel B depicts the lysosomal stress response in Gaucher cells, characterized by the activation of MITF/TFE family transcription factors, which translocate to the nucleus and drive the expression of lysosomal and stress-response genes. This includes upregulation and secretion of gpNMB, a marker of lysosomal burden. Other secreted biomarkers include CCL18/PARC, chitotriosidase and tartrate-resistant acid phosphatase, and ferritin. gpNMB: glycoprotein nonmetastatic melanoma B, CCL18: CC chemokine ligand 18, PARC: pulmonary activation regulated chemokine, GlcCer: glucosylceramide, GCase: glucocerebrosidase

The underlying complexity of Gaucher disease and its diverse neurological manifestations that include Parkinson’s disease and Lewy body dementia, emphasise the need for effective biomarkers to better understand pathogenesis, guide early diagnosis, predict GD manifestations and monitor treatment efficacy (Fig. 1). Although various blood biomarkers—including chitotriosidase, chemokine ligand-18/pulmonary activation-regulated chemokine (CCL18/PARC), ferritin, angiotensin-converting enzyme (ACE), and tartrate-resistant acid phosphatase (TRAP) are commonly elevated in Gaucher disease (GD) (Fig. 1) but few are monospecific for GD [47–50]. Chitotriosidase is absent in < 5% and reduced in about 30% of the population due to an intragenic 24 bp duplication in the human *CHIT* gene that occurs at polymorphic frequency in European and other populations. Moreover, in some instances the biomarker values overlap with those found in healthy individuals – as expected after successful exposure to full doses of enzyme therapy. Plasma glucosylsphingosine (GlcSph) has been considered the most predictive and sensitive biomarker among the recently established biomarkers hitherto identified [51, 52]. The second most used biomarker is plasma chitotriosidase activity, which shows marked elevation from the defined reference values in the treatment-naive population before the advent of ERT in Western countries [50]. However, chitotriosidase is also subject to wide assay variation with nonlinear kinetics when the widely available 4MU-chitobioside is used as substrate and complicated by a very frequent missense GBA1 enzyme variant. Saturating substrate concentrations can, however, be used with the newly designed substrate 4MU-deoxychitobioside [53].

To date, some studies have investigated biomarkers such as gpNMB in LSD patients [2, 54–56]. gpNMB is an endogenous type 1 transmembrane glycoprotein [57], that directly stimulates lipogenesis in white adipocytes [58]. Glycoprotein NMB is expressed by melanocytes [59], osteoclasts [60], macrophages [61] and dendritic cells [62], and hence it has two names: Glycoprotein Non-metastatic Melanoma Protein B and osteoactivin [55]. There is also a secreted form of gpNMB, although this has not been well characterized [63]. Concentrations of gpNMB have been found to be highly correlated with the accumulation of bioactive lipid substrates such as glucosylsphingosine [64].

A specific type of microglia classified as disease-associated microglia (DAM) are found in neurodegenerative diseases [65]. gpNMB is expressed in DAMs [66, 67] and may play an inflammatory role in the CNS [68]. Although its role in pathogenesis has not been fully clarified, elevated concentrations of gpNMB in plasma have been reported in LSDs including GD [2, 69], Niemann-Pick disease type C [56], Tay-Sachs, Sandhoff [70, 71], Mucopolysaccharidosis Type VII [72] and other diseases, including Alzheimer’s Disease [67], multiple sclerosis [73] and cerebral adrenoleukodystrophy (cALD) [74], as a putative regulator of inflammatory responses.

An established regulator of gpNMB expression is melanogenesis-associated transcription factors MITF [62] and TFEB [75], transcription factors known to regulate the expression of proteins involved in autophagy [76, 77] and lysosome biogenesis [13, 78, 79] (Fig. 1). gpNMB has been associated with endosomal/lysosomal structures in phagocytes overexpressing the protein during specific stress conditions [47, 75]. gpNMB has also been reported to be elevated in the CSF of neuronopathic GD (GD3) patients, and a clear correlation between gpNMB concentrations and disease severity in both human and mouse tissues have been reported [2]. However, exactly how gpNMB might relate to neuronopathic disease in Gaucher remains unclear.

Therefore, in this study we have analysed plasma gpNMB concentrations in a large cohort of patients with GD from the GAUCHERITE biobank [17, 80, 81]. We have explored its potential relationship to disease severity and clinical phenotypes, including neurological, pulmonary, and liver disease, and also if there is any correlation between the analyte and development of monoclonal gammopathy or multiple myeloma. The second aim of this study was to determine plasma concentrations of gpNMB in patients with idiopathic Parkinson’s disease (IPD) in order to discover whether there is an association between gpNMB in patients with *GBA* related PD and their neurological manifestations.

## Materials and methods

### Study cohort

gpNMB was determined in plasma collected from 192 patients with Gaucher disease. Clinical data of GD1 and GD3 patients were obtained from the GAUCHERITE database, which includes records of physical examinations, laboratory, radiological, and molecular data [17]. This was a retrospective cross-sectional study. All samples were collected from different expert centers across the UK, and all patients were receiving treatment at the time of sample collection. To describe the burden of Gaucher disease, bone Marrow Burden Score (BMB), modified Severity Scoring Tool (mSST, a tool for monitoring neurological progression correlation and Gaucher disease), Gaucher disease type 1 Disease Severity Scoring System (GD1–DS3) including three major domains (bone, haematologic and visceral) had been previously calculated and documented for each patient [82, 83].

The diagnosis of Gaucher disease was based on acid β-glucosidase assays in leukocytes with confirmatory molecular analysis of the *GBA1* gene. All patients were receiving ERT or SRT. Plasma gpNMB was also determined in plasma obtained from 49 age-and sex-matched healthy control subjects.

The other samples were obtained from 72 patients with idiopathic PD (IPD) who met the United Kingdom Brain Bank criteria and of whom 11 were heterozygous for pathological *GBA1* variants [84]. Plasma samples from a cohort with PD (BioPark cohort from the Karolinska Institute, Sweden) were analysed retrospectively [85]. The *GBA1* gene was sequenced by pyrosequencing or screened by TaqMan PCR and confirmed by Sanger sequencing for the following mutations: p.N370S, p.E326K, and p.L444P.

### Measurement of gpNMB by ELISA

Glycoprotein NMB concentrations were determined according to the manufacturer’s instructions (http://www.RnDSystems.com/ELISADevelopment) using enzyme-linked immunosorbent assay (ELISA) kits (#DY2550 for measuring human gpNMB) from R&D System (Minneapolis, MN). Briefly, plasma samples were diluted 1:100 with phosphate-buffered saline. The capture antibody was plated in 96-well plates overnight at room temperature. After washing with wash buffer and blocking with reagent diluent, 200 ul of the diluted plasma samples and standards were added in duplicate to the plate and incubated at room temperature for 2 hours, followed by repeated washings with wash buffer, repeating the process two times for a total of three washes, and the addition of 100 μl of the diluted detection antibody according to the manufacturer’s instructions. After 2 hours of incubation and washing, the substrate was added to the plate and measured at 450 and 560 nm SPECTRAmax plate reader to provide a wavelength correction and readings were subtracted at 540 nm from the readings at 450 nm. The aim of this subtraction was to correct for optical imperfections in the plate, which might result in higher and less accurate results. A standard curve was created to analyse by reducing the data using PRISM to generate four parameter logistic (4PL) curve fit.

### Glucosylsphingosine analysis with RP-HPLC

Glucosylsphingosine from plasma was extracted in chloroform: methanol (1:2, v/v) with sonication for 10 min at room temperature. Lipids were purified using SPE NH2 columns (Biotage, #470–0010-A). After elution, GlcSph was labelled with o-Phthalaldehyde (OPA) for 20 min at room temperature in the dark and OPA-labelled lipids were taken for analysis by reverse-phase high-performance liquid chromatography (RP-HPLC). The RP-HPLC system consisted of a VWR Hitachi Elite LaChrom HPLC system with a L-2485 fluorescence detector set at Ex λ340 nm and Em λ455 nm. The solid phase used was a Chromolith Performance RP-18e 100–4.6 HPLC column (Merck, Darmstadt, Germany). Individual sphingosine species were identified by their retention time and quantified by comparison of integrated peak areas with a known amount of OPA-labelled C20 sphingosine standard (Avanti Polar Lipids, Alabama, USA) or OPA-labelled C20 glucosylsphingosine standard (Avanti Polar Lipids, Alabama, USA), respectively.

### Statistical analysis

Demographic and laboratory data of study subjects are expressed as mean ± standard deviation for normal distribution and medians (interquartile range) for non-normally distributed variables. Mean, standard deviation, median, minimum, maximum value frequency, and percentage were used for descriptive statistics. Numeric variables and categorical variables are expressed as numbers and percentages. The distribution of variables was checked with the Kolmogorov-Smirnov test. Laboratory values, radiologic features and severity scores were compared between GD1, GD3, IPD, and healthy control groups with the Student’s t-test and the Wilcoxon-Mann-Whitney rank-sum tests if the data were normally distributed. If not normally distributed, the means of groups were compared using the Mann−Whitney U test. For quantitative data, comparisons of three or more groups, ANOVA was used if the data were normally distributed, and the Kruskal−Wallis test was used if the data were not normally distributed. For qualitative data the chi-square test was used if there were more than three groups. Receiver operating characteristic (ROC) curves were plotted, and the area under the ROC curves was calculated to compare the clinical manifestations and diagnostic ability of gpNMB. The effect level was tested with logistic regression and ROC Curves.

The Spearman rank correlation was used to correlate gpNMB and age, anthropometric features (body mass index, weight), biochemical data including acid β-glucosidase activities, lipids (triglyceride, cholesterol, HDL, LDL), full blood counts (haemoglobin, white blood cell, thrombocyte), bone marrow score and GD1–DS3.

All statistical analyses were processed using the Statistical Package for the Social Sciences (SPSS) version 29 (IBM, Armonk, New York, USA) and GraphPad Prism Version 9.3.1. *p* values less than 0.05 were deemed to be statistically significant.

## Results

Concentrations of gpNMB in plasma from 192 patients (91 males, 101 females) with GD were measured from samples stored in the GAUCHERITE Biobank (located in the Department of Pharmacology, University of Oxford).

One hundred and seventy-two (89.5%) (86 females, 86 males) patients were diagnosed with GD type 1, and 20 patients (10.5%) (15 females, 5 males) with GD Type 3. Seven out of 172 patients with GD1 had been diagnosed with PD (4%). The average age of Gaucher patients was 46.2 ± 17.3 years (range 0.6 - 87). Forty-eight (25%) out of 192 patients with GD were homozygous for GBA1 variants – most frequently p.N370S followed by p.L444P, p.R463C and p.W184R; these accounted for 65% (31 patients), 29% (14 patients), 4% (2 patients) and 2% (1 patient) of the 394 mutant *GBA1* alleles, respectively (Supplementary Table 1).

The second patient group in which gpNMB concentrations were measured was the Biopark cohort of 72 IPD patients, 11 of whom were heterozygous for a known *GBA1* variant (p.E326K); the plasma gpNMB was determined in all 72 (48 males, 24 females). The average age of IPD patients was 62.7 ± 9.2 years (range 41 - 97.0). The average age of patients was 62.3 ± 8.8 years (range 41–75) and all were receiving dopaminergic medication (Supplementary Table 2).

Patient data of Gaucher Disease were compared with forty-nine age and sex-matched healthy controls. Although the median (CI) value of gpNMB in males [125 (53.2 - 290 ng/ml)] was slightly greater than that in the females, [105.5 (60.3 - 226.4 ng/ml)], there was no significant gender-related difference (*p* = 0.637). No significant difference in gpNMB concentrations was found between the age sub-groups of patients with 0 - 18, 19 – 49 and 50 - 80 years (*p* > 0.05). There was no significant difference in the BMI values (*p* > 0.05) and the proportion of patients with a BMI > 25 between the Gaucher and control groups (Table 1).

**Table 1:** Characteristics of patients with Gaucher disease and healthy control subject

		Healthy Controls	Gaucher Disease	p	
		Mean ± sd	Mean ± sd		
Age (years)		50.4 ± 15.4	46.2 ± 17.3	0.143	^m^
gpNMB (ng/ml)		35.1 ± 26.4	200.9 ± 240.3	0.0001	^m^
BMI (kg/m^2^)		26.4 ± 5	26.6 ± 6	0.962	^m^
		n	n (GD1+GD3)		
	≤18	4	10 (6 + 4)		^X2^
Age	19- 49	15	94 (81 + 13)	0.068	
	≥50	30	88 (85 + 3)		
Gender	Female	26	101 (86 + 15)	0.954	^X2^
	Male	23	91 (86 + 5)		
BMI (kg/m^2^)	≤25	23	89 (77 + 12)	0.942	^X2^
	> 25	26	103 (95 + 8)		

^m^ Mann-Whitney u test/^X2^ Chi-square test were used to compare variables between Gaucher Disease patients and healthy controls. n: number of patients. GD1: Gaucher Disease Type 1, GD3: Gaucher Disease Type 3, BMI: Body Mass Index

Plasma concentrations of gpNMB in the GD patients receiving treatment were significantly elevated (5.6-fold), with a mean level of 200.9 (range 9.8 - 1643 ng/ml) compared with the matched samples from healthy control subjects (mean 35.1, range 10.1 - 125 ng/ml*, p* < 0.001).

When comparing the median (CI) gpNMB of 20 patients with GD3 [115 (78.48 - 278.9 ng/ml) and 172 patients with GD1 [107 (163.4 - 239.2 ng/ml)], no significant difference was found between these two groups (*p*=0.215) nor between GD1-PD compared with GD1 and GD3 patients (*p*>0.05) (Table 2).

**Table 2:** Comparison of gpNMB concentrations among different patient groups

Group	n	Median gpNMB (ng/ml) IQR	*p*^a^	*p*^b^	*p*^c^	*p*^d^	*p*^e^	*p*^f^
Healthy controls	49	23.9 (17.2–49.1)						
Idiopathic Parkinson’s disease	61	23.3 (14.5–48.3)	> 0.05					
*GBA* heterozygous with Parkinson’s Disease	11	13.7 (10.3–23)	> 0.05	> 0.05				
Gaucher disease (Type 1 and 3)	192	107 (54.6–233.4)	< 0.0001	< 0.0001	< 0.0001			
Gaucher with Parkinson’s Disease	7	241.8 (156–386)	< 0.0001	0.0015	0.0001	> 0.05		
Gaucher disease Type 3	20	115 (65.2–183.5)	< 0.0001	< 0.0001	< 0.0001	> 0.05	> 0.05	
Gaucher disease Type 1	165	107 (53.6–251.5)	< 0.0001	< 0.0001	> 0.05	> 0.05	> 0.05	> 0.05

IQR: Interquartile range, *p*-values: Group comparisons were performed as follows: ***p***^***a***^: Healthy controls vs. all other groups, ***p***^***b***^: Idiopathic Parkinson’s disease vs. Gaucher disease (Types 1 and 3) and Gaucher disease with Parkinson’s Disease, ***p***^***c***^: GBA heterozygous individuals vs. Gaucher disease groups and Gaucher with Parkinson’s Disease, ***p***^***d***^: Gaucher disease (Types 1 and 3) vs. Gaucher with Parkinson’s Disease, ***p***^***e***^: Gaucher with Parkinson’s Disease vs. Gaucher Type 1 or Type 3, ***p***^***f***^: Gaucher Type 1 vs. Gaucher Type 3

Plasma gpNMB concentrations were lower in patients with IPD and *GBA* heterozygous IPD patients from the Biopark Cohort compared with patients with GD1, GD3 and GD1 with PD *(p<*0.001*, p<*0.001*, p<*0.001) from the GAUCHERITE Cohort, respectively. No significant differences were found in gpNMB concentrations between patients with IPD and *GBA* heterozygous PD nor between healthy controls compared with IPD and GBA heterozygous PD (Table 2).

Fifty-eight (30.2%) out of 192 patients had undergone splenectomy. Pulmonary manifestations were documented in 29 (15.1%) patients who were symptomatic. Twenty patients with GD3 and 9 with GD1 had one of the following pulmonary manifestations: pulmonary haemorrhages requiring therapeutic embolization, isolated pulmonary hypertension, asthma, interstitial lung disease, chronic obstructive pulmonary disease, bronchiectasis, pneumothoraces, and pulmonary fibrosis. However, routine pulmonary screening (e.g., chest radiography, computed tomography, or pulmonary function tests) was not performed in all patients, particularly in asymptomatic individuals.

Liver involvement, characterized by fibrosis and cirrhosis, was identified in 14 patients (7.2%) within the cohort, including 5 individuals diagnosed with GD3. Of 14 (7.2%) patients with gammopathy, 10 were diagnosed with monoclonal gammopathy, 3 had multiple myeloma, and one had non-Hodgkin lymphoma (Table 3).

**Table 3:** Clinical characteristics of patients with Gaucher disease

		N
Liver disease		14
Splenectomy		58
Monoclonal Gammopathy		14
Pulmonary disease		29
Parkinson’s disease		7
Treatment	ERT	179
	SRT	13
Treatment duration (mean ± SD): 14.5 ± 7.1 (0.5-29) years		
Dead		17
Alive		175

ERT: Enzyme replacement therapy, SRT: Substrate Reduction Therapy

Compared with healthy subjects, median gpNMB concentrations in the GAUCHERITE Cohort were increased 7, 7, 15 and 17-fold in patients with splenectomy, pulmonary disease, monoclonal gammopathy and liver disease, respectively. Fifty-eight patients who had undergone splenectomy had statistically higher gpNMB concentrations than GD patients with an intact spleen (*p=*0.0012). Patients with pulmonary disease (*n* = 29) had higher plasma gpNMB concentrations compared with those without pulmonary involvement (*n* = 163) (*p* = 0.038). Patients with overt liver disease (*n* = 14) in association with GD also had higher gpNMB concentrations than those without liver disease (*p<*0.05).

GD patients with monoclonal gammopathy including those with B-cell malignancy, (3 patients with plasma cell myeloma, 1 patient with B cell non-Hodgkin lymphoma, and 10 patients with monoclonal gammopathy) (*n* = 14) showed higher gpNMB concentrations in plasma compared with GD patients without monoclonal gammopathy (*p=*0.0004) (Table 4). Within the gammopathy group, one patient had plasma cell myeloma and pulmonary disease, and 3 had monoclonal gammopathy and liver disease. Two of the 14 patients with the highest gpNMB values had pulmonary disease, liver disease, and monoclonal gammopathy. Additionally, one of these patients had pancreatic carcinoma.

**Table 4:**
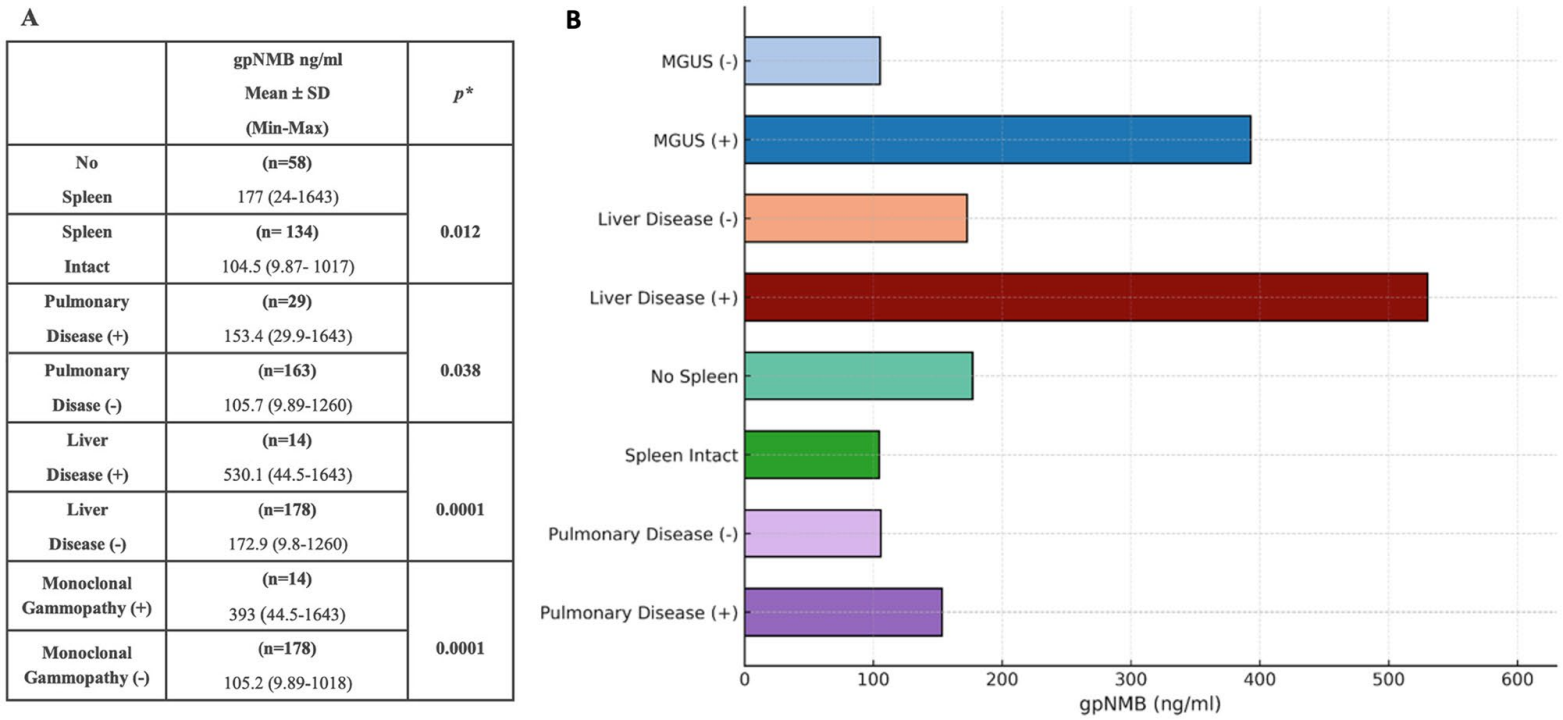
gpNMB concentrations in patients with various clinical conditions (**A**) gpNMB concentrations in patients with and without Spleen, Pulmonary Disease, Liver Disease, and Monoclonal Gammopathy (**B**) Distribution of patients with various clinical conditions in bar chart

MGUS: Monoclonal gammopathy of undetermined significance

**Fig. 2:**
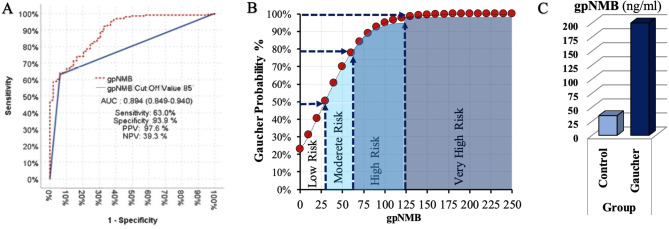
(**A**) receiver operating characteristic curve for gpNMB in Gaucher disease subjects and healthy controls. Figure shows the receiver operating characteristic curve for gpNMB compared with control group with the area under the curve (AUC) = 0.894. (**B**) probability chart for the diagnosis of Gaucher disease based on gpNMB concentrations. (**C**) comparison of gpNMB concentrations in Gaucher disease subjects and healthy controls depicted as a box plot

In discriminating between patients in the control group and the Gaucher group, the gpNMB value showed a good diagnostic performance [Area Under the Curve (AUC) 0.894 (CI,0.849- 0.940)); sensitivity 63.0%; positive predictive value 97.6%; specificity 93.9%, and negative predictive value 39.3%] (Fig. 2A).

Due to the increased probability of GD diagnosis as the gpNMB increases, we divided Gaucher Disease probability into four groups of low ( < 25 ng/ml), medium (25–50 ng/ml), high (65–125 ng/ml) and very high (≥125 ng/ml) probability according to gpNMB calculations. The measurements > 125 ng/ml suggested GD diagnosis with a very high probability ( > 98%) (Figs. 2B, 2C).

**Fig. 3:**
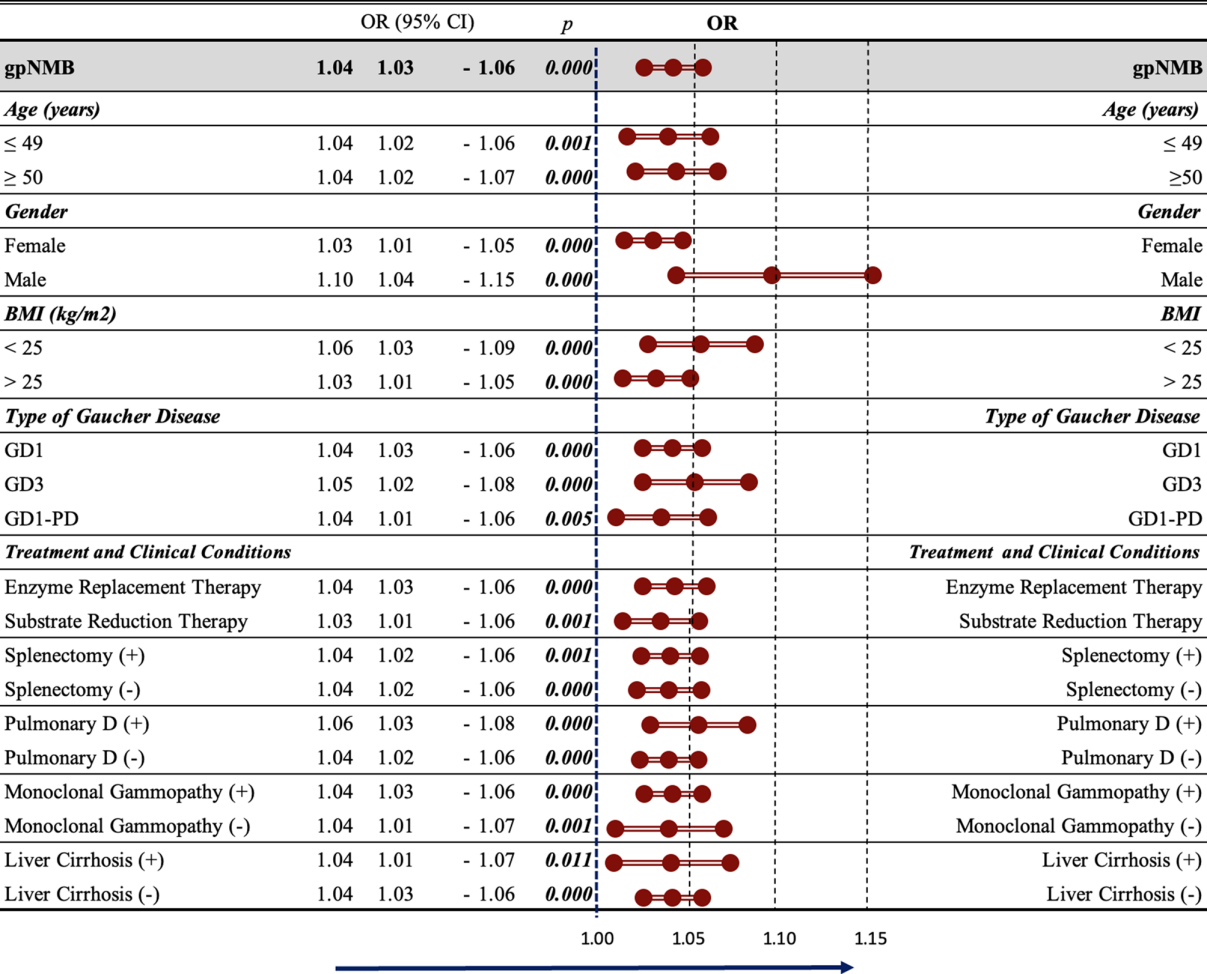
Effectiveness of gpNMB concentrations in distinguishing Gaucher disease subjects based on demographics and clinical characteristics. CI: confidence interval, D: disease

Glycoprotein NMB concentrations were significantly elevated in the treated Gaucher patients (*p<*0.05) [OR 1.04 (1.03–1.06)] compared with healthy control subjects. When comparing all groups with healthy controls, the gpNMB value added discriminatory weight to classifying Gaucher patients in various subgroups: in both the ≤49 age group and the ≥50 age group (*p<*0.05), in both the female and male groups (*p <*0.05), and in both the < 25 BMI and > 25 BMI groups (*p<*0.05). The gpNMB concentrations were also significantly robust in distinguishing GD1, GD3, and GD1-PD from the healthy controls (*p* < 0.05). Furthermore, although gpNMB values clearly distinguish Gaucher patients from healthy controls within each treatment group (ERT or SRT) (*p* < 0.05), it should be noted that some treated patients had plasma gpNMB concentrations that were only slightly above the range of healthy control values. Additionally, in both splenectomized and non-splenectomized groups, pulmonary disease and non-pulmonary disease groups, groups with and without monoclonal gammopathy, and groups with and without liver disease, the gpNMB value was effective in distinguishing Gaucher patients from healthy controls (*p* < 0.05) (Fig. 3). IgG and A concentrations were moderately and weakly correlated with gpNMB, respectively *(rho:* 0.408*, rho:* 0.369*) (p<*0.001*, p<*0.001) (Supplementary Figure 1).

All patients were receiving specific treatment for Gaucher disease: ERTs (179, 93.2%) or SRTs (13, 6.8%). At the time of the study, the mean duration of treatment was 14.6 ± 6.9 years (range: 1–29 years) for patients receiving ERT, and 10.1 ± 7.5 years (range: 6 months–23 years) for those receiving SRT.

The average score of GD1–DS3 was 2.63 ± 1.39 (0.2–6.27) in GD Type 1 patients. Bone marrow burden scores and GD1–DS3 were weakly correlated with gpNMB concentrations *(rho:* 0.337*, rho:* 0.317*) p<*0.001*, p<*0.001) respectively (Supplementary Fig. 2A, 2B) indicating that ordinal measurement was a weak association with these measures of disease severity in patients principally in the late treatment phase of the illness. In patients (*n* = 23) whose treatment duration was 5 years or less than 5 years, a moderate negative correlation was found between gpNMB concentration and treatment duration *(rho=*-0.470*, p=*0.024) (Supplementary Fig. 2C). However, the treatment duration of GD patients of 10 years or more did not correlate with the putative gpNMB biomarker *(rho=*0.114*, p=*0.252).

**Fig. 4:**
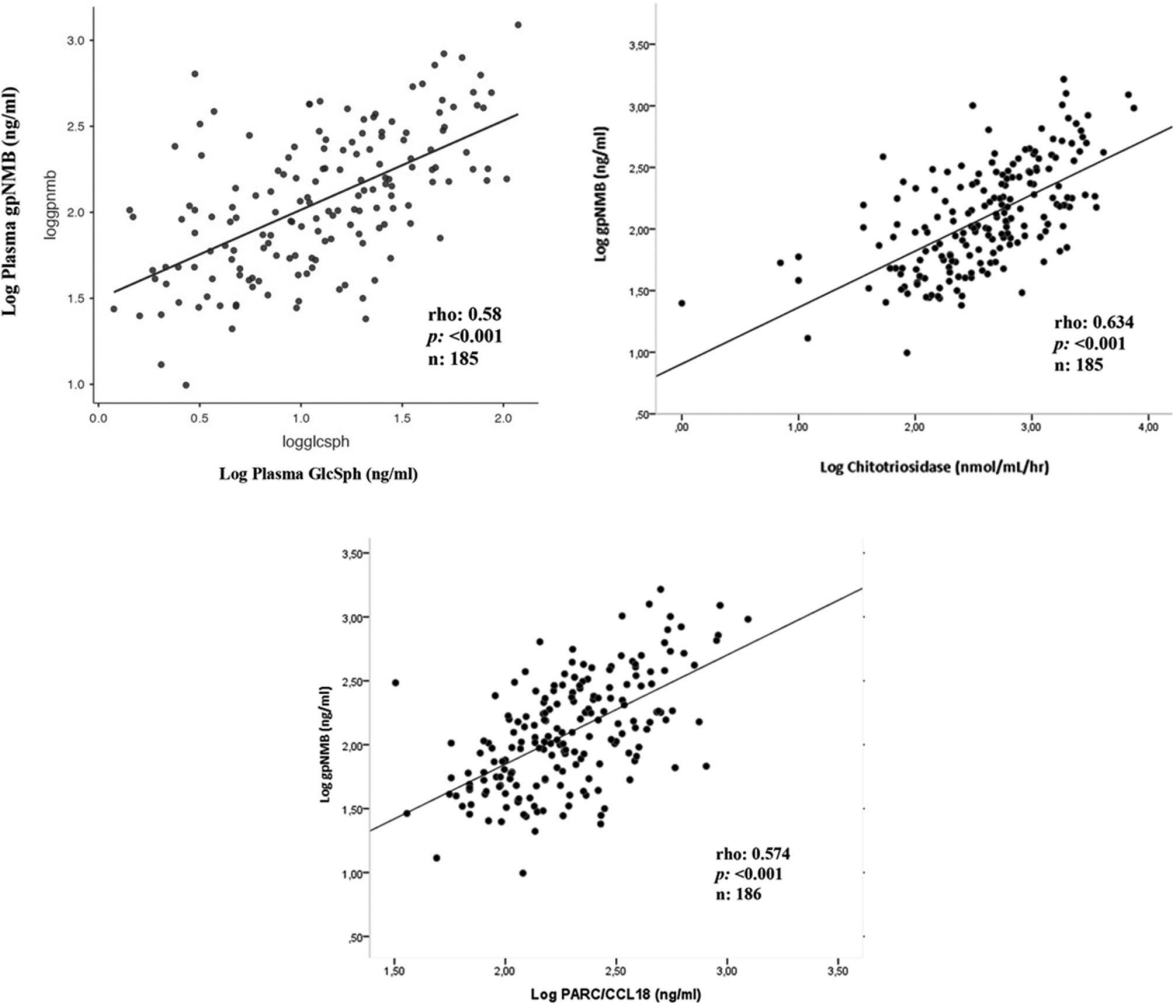
Correlation of GpNMB, Chitotirosidase and CCL18 with plasma GlcSph

Given the established role of glucosylsphingosine (GlcSph) as a key pathogenic molecule in Gaucher disease, its association with plasma gpNMB concentrations was investigated. A statistically significant positive correlation was identified between gpNMB and GlcSph, Chitotriosidase and CCL18 concentrations, respectively (rho = 0.584, rho = 0.634, rho = 0.574, *p* < 0.001) (Fig. 4).

**Fig. 5:**
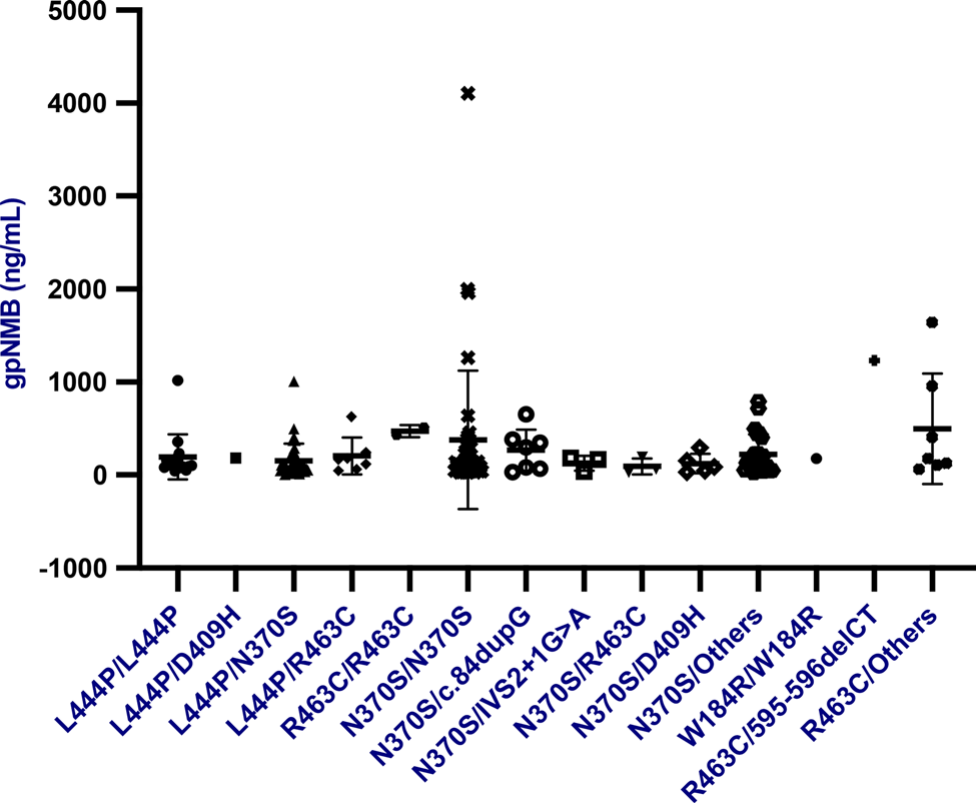
Distribution of gpNMB concentrations in patients with Gaucher disease according to disease causing mutations in the *GBA* gene

For completeness, plasma gpNMB concentrations in these subgroups of Gaucher disease in the era of treatment showed no statistical association with the following parameters: body mass index, current age, mSST of GD3 patients, liver and spleenvolumes, GCase activity, leukocyte or platelet count, haemoglobin concentration, triglyceride, cholesterol, High-Density Lipoprotein (HDL), Low-Density Lipoprotein (LDL), Aspartate Aminotransferase (AST), Alanine Aminotransferase (ALT), Alkaline Phosphatase (ALP) and Gamma-glutamyl transpeptidase (GGT), liver and spleen volumes determined by MRI or CT (magnetic resonance imaging or computerized X-ray tomography) (Supplementary table 3). Of note but not unexpected in the late treatment era, no significant differences were found between mutation groups and plasma gpNMB concentrations (Fig. 5).

No correlation was found between plasma gpNMB concentrations and the Parkinson’s Disease Rating Scales (Total MDS_UPDRS, MDS_UPDRS_III, NMSQ, MOCA, Hoehn and Yahr Scale) or the age of patients with IPD from the Biopark Cohort.

## Discussion

An ideal biochemical biomarker should be readily, reliably, and inexpensively determinable in biological samples that are easily accessed in the clinical setting. The candidate analyte should have high specificity for the disease under study and a wide response range, beyond reference values in healthy and disease control groups; the measured values should vary according to accepted parameters of pathological severity. The biomarker may serve as a diagnostic arbiter of the disease of interest and would ideally also reflect salutary responses to specific therapies. It would thus be invaluable for disease monitoring and thereby contribute valuable information used to predict long-term therapeutic outcomes. The combined assessment of acid β-glucosidase enzymatic activity and *GBA1* mutation analysis is broadly recognized as the standard diagnostic approach for Gaucher disease. Nonetheless, the presence of non-specific clinical features, limitations in enzymatic assay accuracy, and the detection of variants of uncertain significance (VUS) in genetic testing continue to constitute significant sources of diagnostic uncertainty. Several biomarkers have been developed for the diagnosis and clinical monitoring of GD but as yet none has met the criteria for formal registration by regulatory bodies [86, 87].

Increased gpNMB concentrations in plasma from treatment-naive and treated Gaucher patients have been reported to be elevated 15-fold and 10-fold compared with healthy control subjects, respectively [54, 69]. In the current GAUCHERITE cohort, an average of 5.6-fold (4.7- 6.7) increase of plasma gpNMB concentrations was found in Gaucher patients receiving treatment compared with healthy control subjects. Plasma concentrations of gpNMB were highly correlated with established biomarkers, including chitotriosidase activity, CCL-18/PARC concentrations, and the activity of ACE. Elevation of these biomarkers in the bloodstream has been attributed to the presence of pathological macrophages that undergo alternative-type activation (M2 activation) in the context of Gaucher disease [55, 88].

Chitotriosidase, CCL-18/PARC, and ACE in some ways reflect the singular inflammatory process that characterizes this disease. However, the extent to which these molecules contribute to the pathogenesis of Gaucher disease or in part compensate for the consequential effects of the dysregulated glycosphingolipid metabolism, has yet to be resolved. Expression of these biomarkers in patients with neurological Gaucher disease (GD3) did not differ statistically from the values obtained in GD1 (or apparently ‘non-neuronopathic’) patients [51].

Glucosylsphingosine, the deacylated metabolite of glucosylceramide (GlcCer), has emerged as a reliable biomarker for GD activity and therapeutic monitoring. It originates from the accumulation of GlcCer, which is deacylated by lysosomal acid ceramidase in circumstances when acid β-glucosidase is deficient in GD patients [89]. In the plasma of untreated GD1 patients, GlcSph concentrations were elevated by more than 200-fold on average when compared with healthy controls [52, 82]. Glucosylsphingosine is readily measurable in clinical samples with high analytical sensitivity and has demonstrated robust diagnostic and prognostic value, including correlation with chitotriosidase activity and CCL-18-PARC concentrations and declines after institution of ERT or SRT [82, 90]. Elevated glucosysphingosine (GlcSph) is reported in the grey matter and cerebellum of neuronopathic GD patients, and in found in excess in the cerebrospinal fluid of patients with GD1. The latter has been proposed as a possible early biomarker of PD in this population [91, 92]. In the present cohort, plasma gpNMB concentrations were found to be correlated with GlcSph concentrations (rho = 0.584, *p* < 0.001), in line with previous findings [54].

Neuropathology in Gaucher disease may be quantified using gpNMB, which has been proposed as a new useful biomarker due to its expression in activated microglia [68, 93]. gpNMB is implicated during periods of lysosomal stress induced in part by macroautophagy [55]. High concentrations of gpNMB in the cerebrospinal fluid of three GD3 patients and GD3 mice have previously been described [2, 94].

Patients recruited to this study cohort had received macrophage-targeted enzyme therapy irrespective of their neurological status. Nonetheless, plasma gpNMB concentrations were markedly increased, indicating residual activity of their Gaucher disease. Although a mean 4.5 and 6-fold increase beyond the average plasma concentration of gpNMB in healthy control subjects was found, these values did not differ significantly between GD1 and GD3 patients respectively; there was also no significant difference between these patient subgroups, and plasma gpNMB concentrations and mSST scores were not correlated.

There is evidence that Parkinson’s disease is strongly linked to disordered lysosomal function, and while the suggested relationship of the neurological disease to the metabolism of sphingolipids is unclear, apart from increasing age, mutations in *GBA,* the casual gene in Gaucher disease, are the strongest genetic determinants of this neurodegenerative syndrome in populations across the world [95–99]. The risk of PD in Gaucher patients with homozygous or compound heterozygous mutations in *GBA1* is 20-fold higher than in the general population [100, 101]. The risk of PD is also approximately five-fold greater in heterozygous carriers of mutations in *GBA1* compared with non-carriers at all ages [96, 100, 102]. Of note, this phenomenon includes missense mutations in the *GBA1* gene that are widespread and which, if present in homozygosity or in compound heterozygosity with known mutant alleles linked to Gaucher disease, neither cause Gaucher disease nor functional deficiency of catalytic activity of β-glucosylceramidase towards glucosylceramides. Furthermore, altered expression of the *GPNMB* gene has been reported to be greatest in the remaining dopaminergic neurons in the brains of patients with late-stage PD [103]; the abundance of gpNMB protein in the substantia nigra is also elevated [68]. With this in mind, no significant differences were identified in the GAUCHERITE cohort, between plasma gpNMB concentrations in patients with idiopathic Parkinson’s disease and Parkinson’s disease in *GBA1* heterozygotes relative to healthy control subjects; indeed the only group criterion for defining a statistically raised plasma concentration was individuals with a diagnosis of Gaucher disease. Although only 7 patients with a diagnosis of Gaucher disease had PD at the time of sampling, it is perhaps not surprising that plasma gpNMB concentrations were significantly elevated in patients affected by Gaucher disease and PD when compared with patients suffering from either idiopathic Parkinson’s disease or Parkinson’s disease associated with heterozygous *GBA*1 mutations. This is because gpNMB is a secreted product of macrophages- cells that are chiefly distributed in peripheral organs and viscera and thus the main source of circulating gpNMB. Mean plasma concentrations of gpNMB in Gaucher patients with PD were slightly but not significantly greater than in GD3 and GD1 patients without identified Parkinson’s disease. Several patients with Parkinson’s and Gaucher diseases also had liver and pulmonary disease, which are likely to be an additional source of the circulating plasma gpNMB protein. Whether gpNMB might be explored as a potential new biomarker for predicting the risk of developing PD in the GD1 population must, on the basis of the current findings, remain speculative.

We found that during the first 5 years of specific therapy, plasma gpNMB concentrations were inversely correlated with the duration of the treatment. After 10 years of therapy, no correlation was found between gpNMB concentrations and treatment duration, perhaps reflecting the impact of long-term stabilization of disease and secretion of the protein from pathological macrophages and cognate cells sequestered at peripheral sanctuary sites in tissue locations that are not readily accessed by circulating therapeutic ERT proteins.

We have shown that gpNMB and cysteine proteinases such as cathepsins B, K, S, and CCL-18/PARC, are highly expressed in spleens of GD patients and GD modelled in mice [49, 54, 69]. As in the cohort here, patients who had undergone splenectomy generally exhibited higher plasma concentrations of gpNMB compared to those with intact spleens. This finding may be associated with the fact that gpNMB is particularly expressed in macrophages and monocytes, which are widely distributed in organs affected by Gaucher disease [54].

gpNMB expression appears to be highly regulated: granulocyte-macrophage colony-stimulating factor and macrophage-colony stimulating factor both stimulate monocytes and induce secretion of gpNMB [104]. gpNMB is significantly increased in several unrelated diseases – notably patients with inflammatory respiratory diseases such as chronic obstructive pulmonary disease patients [105]. gpNMB present in the fibrotic extracellular matrix was shown to originate from macrophages [106]. Administration of gpNMB-neutralizing antibodies reduced the severity of pulmonary fibrosis. Therefore, high circulating or local gpNMB release might be responsible for pathological changes in extracellular matrix and the development of tissue fibrosis, which is a feature of Gaucher disease infiltration in the lung, liver, and spleen [106, 107]. In support of this notion we found that plasma gpNMB concentrations of patients with pulmonary infiltration were greater than those in other Gaucher patients. It has been reported that serum gpNMB concentrations were higher in patients with nonalcoholic steatohepatitis compared with simple steatosis. Additionally, it has been found that serum gpNMB concentrations in other clinical contexts correlated with the severity of liver fibrosis [108]. Similarly, in our study, gpNMB concentrations of patients with liver disease, including liver fibrosis and liver cirrhosis, were statistically greater than in those patients without manifest liver disease.

There remain many unknown properties of gpNMB and its potential role as a biomarker in several disorders has yet to clarified. An increased frequency of multiple myeloma and monoclonal gammopathy or paraproteinaemia has long been recognized in Gaucher patients [19, 109]. Even though gpNMB is considered to be a potent angiogenic factor in gammopathies and multiple myeloma, the extent to which if any, it contributes to the pathogenesis of the cancer and its progression is unknown. In a previous study, gpNMB in the cohort of monoclonal gammopathy of undetermined significance (MGUS) in the general population exhibited the greatest expression compared with the control group [110]. In the present cohort, the concentrations of gpNMB were statistically higher in GD patients with MGUS compared with patients without MGUS.

Pathological accumulation of glucosylsphingosine and glucosylceramides are stongly implicated in the chronic macrophage inflammation and may contribute to the polyclonal production of immunoglobulins. [64, 111–115]. In the presented cohort, IgG and IgA but not IgM levels correlated with gpNMB, which is consistent with chronic inflammation in GD despite treatment.

Expressed in macrophages and hypertrophied adipocytes, gpNMB was also reported to play a crucial role during obesity in white adipose tissue inflammation, which is critical in obesity-related metabolic disorders in mouse models [116]. However, in our study, no correlation was found between plasma gpNMB concentrations and the body mass index of Gaucher patients. In this cohort, the two most frequent mutations in the *GBA1* gene were p.N370S and p.L444P. In general, patients with homozygous p.N370S mutations tend to have a milder GD1 phenotype and do not develop neuropathic complications other than Parkinson’s disease or Lewy body dementia. In contrast, homozygous individuals for p.L444P tend to have a severe GD3 phenotype as in the present cohort [117, 118]. However, genotype-phenotype heterogeneity has been shown in mutations of the *GBA1* gene with a distinctive clinical variability previously demonstrated between monozygotic twins with the same genotype, likely due to an interaction between modifier genes and environmental factors including imprinting [119, 120]. In this context and in patients late into the treatment of their illness we noted there was considerable variation in gpNMB and a wide distribution across the different subtypes, especially where there are limitations to the differentiation of GD3 from GD1. With regard to the pleiotropic nature of the disease, we contend that gpNMB, may ultimately assist in supporting the deep phenotyping of the disorder and provide a more refined means for predicting the course of the illness.

Glycoprotein NMB appears to have significant potential for in-depth evaluation of pathogenesis across distinct clinical trajectories of disease. Quantification of gpNMB in patient samples collected before starting treatment and at intervals, thereafter, may prove more informative, in particular to explore biomarker changes that preferentially associate with therapeutic responses in disparate organ systems.

## Conclusion

Overall, plasma gpNMB concentrations show potential for detecting fibrotic liver and pulmonary disease and MGUS. The association between elevated gpNMB concentrations and these clinical features in Gaucher disease warrants further investigation. Importantly, gpNMB measurements were performed in patients already receiving treatment, and response to therapy may vary across GD subtypes. Therefore, the apparent similarity in gpNMB concentrations between GD1 and GD3 patients does not necessarily indicate comparable disease burden, particularly in the absence of pre-treatment baseline data. Conversely, the presence of markedly elevated gpNMB concentrations in individual treated patients—especially those with liver disease and monoclonal gammopathy—deserves further exploration. With additional studies, monitoring gpNMB may aid in clinical stratification of GD patients with a confirmed genetic diagnosis.

## Electronic supplementary material

Below is the link to the electronic supplementary material.

Supplementary Material 1

## Data Availability

The datasets used in this study may be available with restrictions to some details (for patient confidentiality) from the corresponding author on reasonable request.

## Abbreviations

ACE: Angiotensin-converting enzyme
CCL18: Chemokine ligand-18 tartrate-resistant acid phosphatase (TRAP)
CI: Confidence interval
MGUS: Monoclonal gammopathy of undetermined significance
ERT: Enzyme replacement therapy
GD: Gaucher Disease
GD1: Gaucher Disease type 1
GD3: Gaucher Disease type 3
GAUCHERITE: Gaucher Investigative Therapy Evaluation
GD-DS3: Gaucher Disease Type 1 Severity Scoring System
gpNMB: Glycoprotein non-metastatic melanoma protein B
LSD: Lysosomal Storage Disease
NHS: National Health Service
PARC: Pulmonary activation-regulated chemokine
PD: Parkinson’s Disease
SRT: Substrate reduction therapy
SD: Standard deviation
UK: United Kingdom

## Declarations

### Limitations of the study

We acknowledge some limitations to our study. Plasma gpNMB concentrations were analysed in patients who were already on treatment. Since analyses were performed at a specific time for all patients, long-term gpNMB alterations during the disease process were not captured in this study. Additionally, the relatively small number of patients receiving SRT may limit the statistical power to detect subtle differences between treatment modalities.
