# Correction: Glycoprotein non-metastatic melanoma protein B is a biomarker of inflammation in individuals with Gaucher disease: relationship to clinico-pathological subtypes

**DOI:** 10.1186/s13023-025-04143-y

**Published:** 2025-11-17

**Authors:** Sebile Kilavuz, Kerri-Lee Wallom, Ana Catarina Gomes Almeida Augusto Caçote, Aimée Donald, Simona D’Amore, Kathy Page, Chong Yew Tan, Danielle te Vruchte, Andrea Sturchio, Panagiota Tsitsi, Ellen Hertz, Mattias Andréasson, Ioanna Markaki, Per Svenningsson, Timothy M. Cox, Frances M. Platt, the MRC GAUCHERITE Consortium

**Affiliations:** 1 Department of Pharmacology, University of Oxford, Mansfield Road, Oxford, OX1 3QT UK; 2 Division of Pediatric Metabolism and Nutrition, Department of Pediatrics, Marmara University Faculty of Medicine, Istanbul, Turkey; 3 Division of Neurosciences, Faculty of Biology, Medicine & Health, University of Manchester, Manchester, UK; 4 Department of Medicine, Cambridge Lysosomal Storage Disorders Unit, Royal Free Hospital NHS Foundation Trust, University of Cambridge, London, UK; 5 Department of Precision and Regenerative Medicine - Ionian Pole, University of Bari Aldo Moro, Bari, Italy; 6 Department of Medicine, University of Cambridge, Cambridge, UK; 7 Department of Clinical Neuroscience, Karolinska Institute, Stockholm, Sweden

**Correction: Orphanet J Rare Dis 20, 545 (2025)**

10.1186/s13023-025-04054-y

Following publication of the original article [1], the authors reported an error in the names of the author Dr. Ioanna Markaki and members of the MRC Gaucherite consortium T G Hiwot and Derralynn A Hughes.

The full names present in this Correction are correct.

Additionally, an error was reported in the Legend of Table 4. The Figure type should read as “bar chart” instead of “box plot”.

Typing errors have also been corrected.

The original article [1] has been updated.

## References

[1] Kilavuz S, Wallom KL, Caçote AC, et al. Glycoprotein non-metastatic melanoma protein B is a biomarker of inflammation in individuals with Gaucher disease: relationship to clinico-pathological subtypes. Orphanet J Rare Dis. 2025;20:545. 10.1186/s13023-025-04054-y. 41152894 10.1186/s13023-025-04054-y PMC12570663

