# Supplementary material for: Glycoprotein non-metastatic melanoma protein B is a biomarker of inflammation in individuals with Gaucher disease: relationship to clinico-pathological subtypes
Source: Orphanet J Rare Dis. 2025 Oct 28;20:545. doi: 10.1186/s13023-025-04054-y (PMC12570663; doi:10.1186/s13023-025-04054-y)
Supplement: Supplementary file 1 — Supplementary Material 1 [file 13023_2025_4054_MOESM1_ESM.docx]

**Supplementary Table 1.** *GBA1* Genotypes and Phenotype Classification of Study Cohort

| **N^0^** | **Subtype** | **Allele 1** | **Mutation 1** | **Allele 2** | **Mutation 2** |
| --- | --- | --- | --- | --- | --- |
| 1 | GD1 | N370S | c.1226A>G | L444P | c.1448T>C |
| 2 | GD1 | L444P | c.1448T>C | N370S | c.1226A>G |
| 3 | GD1 | L444P | c.1448T>C | N370S | c.1226A>G |
| 4 | GD1 | R463C | c.1504C>T | L444P | c.1448T>C |
| 5 | GD1 | L444P | c.1448T>C | N370S | c.1226A>G |
| 6 | GD1 | R463C | c.1504C>T | N370S | c.1226A>G |
| 7 | GD1 | N370S | c.1226A>G | N370S | c.1226A>G |
| 8 | GD1 | R463C | c.1504C>T | N370S | c.1226A>G |
| 9 | GD3 | D409H | c.1342G>C | L444P | c.1448T>C |
| 10 | GD1 | N370S | c.1226A>G | L444P | c.1448T>C |
| 11 | GD1 | N370S | c.1226A>G | N370S | c.1226A>G |
| 12 | GD1 | R496H | c.1604G>A | N370S | c.1226A>G |
| 13 | GD1 | R463C | c.1504C>T | L444P | c.1448T>C |
| 14 | GD1 | N370S | c.1226A>G | L444P | c.1448T>C |
| 15 | GD1 | D218V | c.653A>T | N370S | c.1226A>G |
| 16 | GD1 | N370S | c.1226A>G | A318D | c.1070C>A |
| 17 | GD1 | L444P | c.1448T>C | N370S | c.1226A>G |
| 18 | GD1 | N370S | c.1226A>G | P182T | c.661C>A |
| 19 | GD1 | N370S | c.1226A>G | N370S | c.1226A>G |
| 20 | GD3 | D409H | c.1342G>C | R463C | c.1504C>T |
| 21 | GD1 | R463C | c.1504C>T | L444P | c.1448T>C |
| 22 | GD1 | D474N | c.1537G>A | N370S | c.1226A>G |
| 23 | GD1 | N370S | c.1226A>G | R120W | c.475C>T |
| 24 | GD1 | N370S | c.1226A>G | N370S | c.1226A>G |
| 25 | GD1 | N370S | c.1226A>G | N370S | c.1226A>G |
| 26 | GD1 | N370S | c.1226A>G | N370S | c.1226A>G |
| 27 | GD1 | N370S | c.1226A>G | N370S | c.1226A>G |
| 28 | GD1 | Y418S | 1370A>C | N370S | c.1226A>G |
| 29 | GD1 | N370S | c.1226A>G | R257Q | c.887G>A |
| 30 | GD1 | RecNcil + RecΔ5 | c.1488T>C/c.1483G>C/  c.1497G>C; c.1263del55 | N370S | c.1226A>G |
| 31 | GD1 | Y22C | c.182A>G | F259L | c.894C>A |
| 32 | GD1 | V394L | c.1297G>T | N370S | c.1226A>G |
| 33 | GD1 | L444P | c.1448T>C | N370S | c.1226A>G |
| 34 | GD1 | c.1388+1G>A | c.1388+1G>A | N370S | c.1226A>G |
| 35 | GD1 | D315H | c.1060G>C | N370S | c.1226A>G |
| 36 | GD1 | N370S | c.1226A>G | N370S | c.1226A>G |
| 37 | GD1 | N370S | c.1226A>G | N370S | c.1226A>G |
| 38 | GD1 | L444P | c.1448T>C | N370S | c.1226A>G |
| 39 | GD1 | N370S | c.1226A>G | L105R | c.431T>G |
| 40 | GD1 | c.1388+1G>A | c.1388+1G>A | N370S | c.1226A>G |
| 41 | GD1 | L444P | c.1448T>C | N370S | c.1226A>G |
| 42 | GD1 | D409H | c.1342G>C | N370S | c.1226A>G |
| 43 | GD1 | IVS 2+1G>A | c.115+1G>A | N370S | c.1226A>G |
| 44 | GD1 | L444P | c.1448T>C | N370S | c.1226A>G |
| 45 | GD1 | Y220C | c.776A>G | N370S | c.1226A>G |
| 46 | GD1 | L444P | c.1448T>C | N370S | c.1226A>G |
| 47 | GD1 | L444P | c.1448T>C | N370S | c.1226A>G |
| 48 | GD3 | L444P | c.1448T>C | L444P | c.1448T>C |
| 49 | GD1 | N370S | c.1226A>G | L444P | c.1448T>C |
| 50 | GD1 | RecNcil + RecΔ5 | c.1488T>C/c.1483G>C/  c.1497G>C; c.1263del55 | F216Y | c.764T > A |
| 51 | GD1 | Y418C | c.1370A>G | N370S | c.1226A>G |
| 52 | GD1 | IVS2+1G>A | c.115+1G>A | R463C | c.1504C>T |
| 53 | GD1 | R463C | c.1504C>T | R463C | c.1504C>T |
| 54 | GD1 | L444P | c.1448T>C | N370S | c.1226A>G |
| 55 | GD1 | c.153-154insTACAGC | c.148-153dupTACAGC | L444P | c.1448T>C |
| 56 | GD1 | L480P | c.1556T>C | N370S | c.1226A>G |
| 57 | GD1 | L444P | c.1448T>C | N370S | c.1226A>G |
| 58 | GD1 | G202R | c.721G>A | R496H | c.1604G>A |
| 59 | GD1 | L444P | c.1448T>C | N370S | c.1226A>G |
| 60 | GD3 | RecNcil recombinant allele | c.1488T>C/c.1483G>C/c.1497G>C | R463C | c.1504C>T |
| 61 | GD1 | L444P | c.1448T>C | N370S | c.1226A>G |
| 62 | GD3 | R463C | c.1504C>T | L444P | c.1448T>C |
| 63 | GD1 | 84GG | c.84dupG | N370S | c.1226A>G |
| 64 | GD1 | R463C | c.1504C>T | N370S | c.1226A>G |
| 65 | GD1 | G377R | c.1246G>C | R463C | c.1504C>T |
| 66 | GD1 | L444P | c.1448T>C | P266R | c.914C>G |
| 67 | GD1 | RecNcil | c.1488T>C/c.1483G>C/  c.1497G>C | N370S | c.1226A>G |
| 68 | GD1 | L444P | c.1448T>C | N370S | c.1226A>G |
| 69 | GD1 | L444P | c.1448T>C | N370S | c.1226A>G |
| 70 | GD1 | N370S | c.1226A>G | c.316_316delC | c.316_316delC |
| 71 | GD1 | N370S | c.1226A>G | I402T + E236K | c.1322T>C + c.1093G>A |
| 72 | GD1 | N370S | c.1226A>G | RecNcil | c.1488T>C/c.1483G>C/  c.1497G>C |
| 73 | GD1 | N370S | c.1226A>G | R257Q | c.887G>A |
| 74 | GD1 | L383P | c.1265T>C | N370S | c.1226A>G |
| 75 | GD1 | N370S | c.1226A>G | RecNcil | c.1488T>C/c.1483G>C/  c.1497G>C |
| 76 | GD1 | N370S | c.1226A>G | N370S | c.1226A>G |
| 77 | GD1 | IVS 2+1G>A +  c.44T>C + c.46A>G | c.115+1G>A; c.44T>C; c.46A>G | N370S | c.1226A>G |
| 78 | GD1 | N370S | c.1226A>G | N370S | c.1226A>G |
| 79 | GD1 | L444P | c.1448T>C | N370S | c.1226A>G |
| 80 | GD3 | L444P | c.1448T>C | L444P | c.1448T>C |
| 81 | GD3 | L444P | c.1448T>C | L444P | c.1448T>C |
| 82 | GD1 | L444P | c.1448T>C | N370S | c.1226A>G |
| 83 | GD1 | N370S | c.1226A>G | N370S | c.1226A>G |
| 84 | GD1 | L444P | c.1448T>C | N370S | c.1226A>G |
| 85 | GD1 | N370S | c.1226A>G | L444P | c.1448T>C |
| 86 | GD3 | L444P | c.1448T>C | L444P | c.1448T>C |
| 87 | GD1 | V394L | c.1297G>T | N370S | c.1226A>G |
| 88 | GD1 | N370S | c.1226A>G | 1VS2+1G>A | c.115+1G>A |
| 89 | GD1 | N370S | c.1226A>G | N370S | c.1226A>G |
| 90 | GD1 | N370S | c.1226A>G | L444P | c.1448T>C |
| 91 | GD1 | N370S | c.1226A>G | 1VS2+1G>A | c.115+1G>A |
| 92 | GD1 | D409H | c.1342G>C | N370S | c.1226A>G |
| 93 | GD1 | N370S | c.1226A>G | N370S | c.1226A>G |
| 94 | GD1 | 84GG | c.84dupG | N370S | c.1226A>G |
| 95 | GD1 | N370S | c.1226A>G | N370S | c.1226A>G |
| 96 | GD1 | R463C | c.1504C>T | N370S | c.1226A>G |
| 97 | GD1 | L444P | c.1448T>C | N370S | c.1226A>G |
| 98 | GD1 | p.84GG | c.84dupG | N370S | c.1226A>G |
| 99 | GD1 | N370S | c.1226A>G | N370S | c.1226A>G |
| 100 | GD1 | 84GG | c.84dupG | N370S | c.1226A>G |
| 101 | GD3 | L444P | c.1448T>C | L444P | c.1448T>C |
| 102 | GD1 | L444P | c.1448T>C | N370S | c.1226A>G |
| 103 | GD1 | N370S | c.1226A>G | N370S | c.1226A>G |
| 104 | GD1 | N370S | c.1226A>G | N370S | c.1226A>G |
| 105 | GD1 | L444P | c.1448T>C | N370S | c.1226A>G |
| 106 | GD1 | L444P | c.1448T>C | N370S | c.1226A>G |
| 107 | GD1 | N370S | c.1226A>G | N370S | c.1226A>G |
| 108 | GD1 | R463C | c.1504C>T | 595-596delCT | c.595_596delCT |
| 109 | GD1 | D380A | c.1256A>C | N370S | c.1226A>G |
| 110 | GD1 | 84GG | c.84dupG | N370S | c.1226A>G |
| 111 | GD1 | L444P | c.1448T>C | N370S | c.1226A>G |
| 112 | GD1 | N370S | c.1226A>G | D409H | c.1342G>C |
| 113 | GD1 | N370S | c.1226A>G | N370S | c.1226A>G |
| 114 | GD1 | N370S | c.1226A>G | N370S | c.1226A>G |
| 115 | GD1 | RecNcil | c.1488T>C/c.1483G>C/  c.1497G>C | N370S | c.1226A>G |
| 116 | GD1 | p.G250V | c.866G>T | N370S | c.1226A>G |
| 117 | GD1 | L444P | c.1448T>C | N370S | c.1226A>G |
| 118 | GD3 | R463C | c.1504C>T | L444P | c.1448T>C |
| 119 | GD1 | c.1388+1G>A | c.1388+1G>A | N370S | c.1226A>G |
| 120 | GD1 | L444P | c.1448T>C | N370S | c.1226A>G |
| 121 | GD1 | H255Q/D409H complex allele | c.882T>G; c.1342G>C | N370S | c.1226A>G |
| 122 | GD1 | D409H | c.1342G>C | N370S | c.1226A>G |
| 123 | GD1 | p.84GG | c.84dupG | N370S | c.1226A>G |
| 124 | GD1 | RecΔ5 | c.1263del55 | N370S | c.1226A>G |
| 125 | GD1 | N370S | c.1226A>G | N370S | c.1226A>G |
| 126 | GD1 | RecNcil | c.1488T>C/c.1483G>C/  c.1497G>C | N370S | c.1226A>G |
| 127 | GD3 | L444P | c.1448T>C | L444P | c.1448T>C |
| 128 | GD1 | R496H | c.1604G>A | 84GG | c.84dupG |
| 129 | GD1 | R120Q | c.476G>A | N370S | c.1226A>G |
| 130 | GD1 | R463C | c.1504C>T | other | other |
| 131 | GD1 | 84GG | c.84dupG | N370S | c.1226A>G |
| 132 | GD1 | N370S | c.1226A>G | D409H | c.1342G>C |
| 133 | GD1 | L444P | c.1448T>C | N370S | c.1226A>G |
| 134 | GD3 | L444P | c.1448T>C | L444P | c.1448T>C |
| 135 | GD1 | L444P | c.1448T>C | N370S | c.1226A>G |
| 136 | GD1 | N370S | c.1226A>G | N370S | c.1226A>G |
| 137 | GD1 | L444P | c.1448T>C | N370S | c.1226A>G |
| 138 | GD1 | R120Q | c.476G>A | N370S | c.1226A>G |
| 139 | GD1 | 84GG | c.84dupG | N370S | c.1226A>G |
| 140 | GD1 | R359X | c.1192C>T | R496H | c.1604G>A |
| 141 | GD3 | R262G | c.901C>G | RecNcil | c.1488T>C/c.1483G>C/ |
| 142 | GD1 | L444P | c.1448T>C | N370S | c.1226A>G |
| 143 | GD1 | L444P | c.1448T>C | N370S | c.1226A>G |
| 144 | GD1 | L105R | c.431T>G | N370S | c.1226A>G |
| 145 | GD1 | N370S | c.1226A>G | N370S | c.1226A>G |
| 146 | GD3 | R463C | c.1504C>T | L444P | c.1448T>C |
| 147 | GD1 | W184R | c.667T>C | W184R | c.667T>C |
| 148 | GD1 | R463C | c.1504C>T | L444P | c.1448T>C |
| 149 | GD1 | F216Y | c.764T>A | L444P | c.1448T>C |
| 150 | GD1 | R463C | c.1504C>T | L444P | c.1448T>C |
| 151 | GD1 | L444P | c.1448T>C | N370S | c.1226A>G |
| 152 | GD3 | L444P | c.1448T>C | L444P | c.1448T>C |
| 153 | GD1 | N370S | c.1226A>G | N370S | c.1226A>G |
| 154 | GD1 | N370S | c.1226A>G | L66P | c.314T>C |
| 155 | GD1 | R463C | c.1504C>T | R463C | c.1504C>T |
| 156 | GD1 | N370S | c.1226A>G | N370S | c.1226A>G |
| 157 | GD1 | N370S | c.1226A>G | N370S | c.1226A>G |
| 158 | GD1 | N370S | c.1226A>G | N370S | c.1226A>G |
| 159 | GD1 | H255Q + D409H | c.1342G>C; c.882T>G | N370S | c.1226A>G |
| 160 | GD3 | L444P | c.1448T>C | L444P | c.1448T>C |
| 161 | GD1 | N370S | c.1226A>G | N370S | c.1226A>G |
| 162 | GD1 | N370S | c.1226A>G | T323I | c.1085C>T |
| 163 | GD3 | L444P | c.1448T>C | L444P | c.1448T>C |
| 164 | GD3 | L444P | c.1448T>C | E233D | c.816A>C |
| 165 | GD3 | L444P | c.1448T>C | L444P | c.1448T>C |
| 166 | GD1 | L444P | c.1448T>C | N370S | c.1226A>G |
| 167 | GD1 | N462K | c.1503C>G | N370S | c.1226A>G |
| 168 | GD1 | N370S | c.1226A>G | L444P | c.1448T>C |
| 169 | GD1 | R120W | c.475C>T | N370S | c.1226A>G |
| 170 | GD3 | L444P | c.1448T>C | L444P | c.1448T>C |
| 171 | GD1 | N370S | c.1226A>G | c.1249_1251 del TGG | c.1249_1251 del TGG |
| 172 | GD1 | N370S | c.1226A>G | RecA456P (L444P + A456P) | c.1448T>C/c.1483G>C |
| 173 | GD1 | N370S | c.1226A>G | R131C | c.508C>T |
| 174 | GD1 | RecNcil (L44P+A456P+V460V) | c.1448T>C,  c.1483G>C, c.1497G>C | N370S | c.1226A>G |
| 175 | GD1 | L444P | c.1448T>C | N370S | c.1226A>G |
| 176 | GD1 | L444P | c.1448T>C | N370S | c.1226A>G |
| 177 | GD3 | R496C | c.1603C>T | R463C | c.1504C>T |
| 178 | GD1 | N462K | c.1503C>G | R463C | c.1504C>T |
| 179 | GD1 | L444P | c.1448T>C | N370S | c.1226A>G |
| 180 | GD1 | F397S | c.1307T>C | c.334_338delcagaa | c.334_338delcagaa |
| 181 | GD1 | RecA456P (L444P + A456P) | c.1448T>C c.1483G>C | N370S | c.1226A>G |
| 182 | GD1 | IVS2+1G>A | c.115+1G>A | N370S | c.1226A>G |
| 183 | GD3 | L444P | c.1448T>C | L444P | c.1448T>C |
| 184 | GD1 | N370S | c.1226A>G | N370S | c.1226A>G |
| 185 | GD1 | D24Y | c.187G>T | N370S | c.1226A>G |
| 186 | GD3 | L444P | c.1448T>C | L444P | c.1448T>C |
| 187 | GD1 | RecNcil | (L444P + A456P + V460V) | N370S | c.1226A>G |
| 188 | GD1 | RecΔ5 | c.1263-1317del55 | N370S | c.1226A>G |
| 189 | GD1 | L444P | c.1448T>C | N370S | c.1226A>G |
| 190 | GD1 | *N370S* | *c.1226A>G* | *L444P* | *c.1448T>C* |
| 191 | GD1 | N370S | c.1226A>G | RecNcil | (L444P + A456P+V460V) |
| 192 | GD1 | N370S | c.1226A>G | L444P | c.1448T>C |

GD1: Gaucher Disease Type 1, GD3: Gaucher Disease Type 3

**Supplementary Table 2** Demographic Features of individuals with Idiopathic Parkinson’s disease and Healthy Controls from Biopark Cohort

|  |  | | Min-Max | | | | Median | Mean ± SD / n-% | | |
| --- | --- | --- | --- | --- | --- | --- | --- | --- | --- | --- |
| **Age (years)** | | 41 | | | - | 97 | 64 | 62.7 | ± | 9.2 |
| **Gender** | **Female** |  | | |  |  |  | 38 |  | 41% |
|  | **Male** |  | | |  |  |  | 54 |  | 59% |
| **Idiopathic Parkinson’s disease group** | | | |  |  |  |  | 72 |  | 79.70% |
| **Healthy controls** | | | |  |  |  |  | 20 |  | 20.30% |
| **Idiopathic Parkinson’s disease group** | | | |  |  |  |  | 72 |  | 100% |
| *GBA* Heterozygous Idiopathic Parkinson’s disease | | | |  |  |  |  | 11 |  | 15% |
| **gpNMB (ng/ml)** | | 4 | | | - | 170.9 | 24.7 | 32.8 | ± | 24.7 |

**Supplementary Table 3.** gpNMB correlation with clinical and laboratory data

|  |  | **gpNMB**  ng/ml |
| --- | --- | --- |
| **Age**  (years) | ***r*** | 0.047 |
|  | ***p*** | 0.527 |
| **Body Mass Index**  (kg/m^2^) | ***r*** | -0.078 |
|  | ***p*** | 0.283 |
| **B Glucosidase**  (nmol/h/mg) | ***r*** | 0.025 |
|  | ***p*** | 0.788 |
| **mSST of GD3 patients** | ***r*** | -0.133 |
|  | ***p*** | 0.600 |
| **WBC** /mm^3^ | ***r*** | 0.083 |
|  | ***p*** | 0.253 |
| **Hb** g/dl | ***r*** | -0.056 |
|  | ***p*** | 0.438 |
| **Plt** /mm^3^ | ***r*** | 0.072 |
|  | ***p*** | 0.328 |
| **Triglyceride**  mmol/L | *r* | 0.061 |
|  | *p* | 0.554 |
| **Cholesterol**  mmol/L | ***r*** | 0.035 |
|  | ***p*** | 0.727 |
| **HDL Cholesterol**  mmol/L | ***r*** | -0.081 |
|  | ***p*** | 0.424 |
| **LDL Cholesterol**  mmol/L | ***r*** | 0.140 |
|  | ***p*** | 0.178 |
| **AST**  U/L | ***r*** | 0.095 |
|  | ***p*** | 0.250 |
| **ALT**  U/L | ***r*** | -0.087 |
|  | ***p*** | 0.233 |
| **ALP**  U/L | ***r*** | 0.094 |
|  | ***p*** | 0.198 |
| **GGT**  U/L | ***r*** | 0.123 |
|  | ***p*** | 0.132 |
| **Liver Volume** | ***r*** | 0.045 |
|  | ***p*** | 0.655 |
| **Spleen Volume** | ***r*** | 0.202 |
|  | ***p*** | 0.107 |

mSST: modified Severity Scoring Tool, HDL: High-Density Lipoprotein, LDL: Low-Density Lipoprotein, AST: Aspartate Aminotransferase, ALT: Alanine Aminotransferase, ALP: Alkaline Phosphatase, GGT: Gamma-glutamyl transpeptidase.


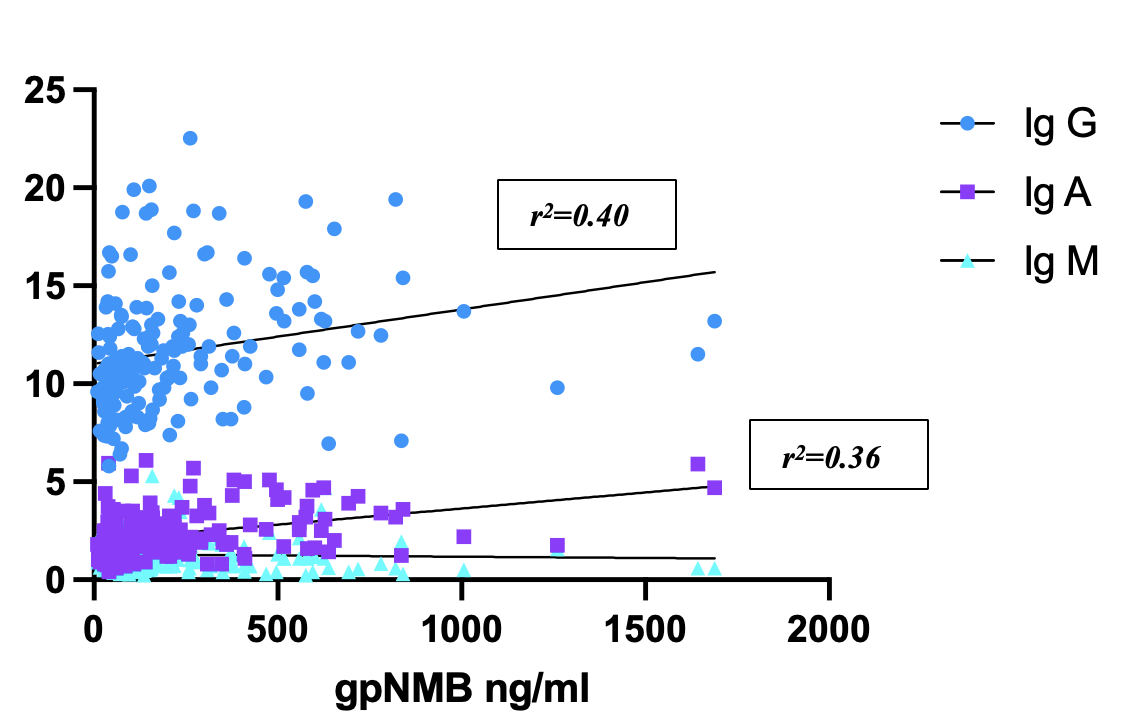


**Serum Immunoglobulin Levels (g/L)**

**Supplementary Fig. 1** Correlation of gpNMB with Immunoglobulin G, A *(p<*0.001*, p<*0.001*)* and M.

Ig: Immunoglobulin, gpNMB: Glycoprotein nonmetastatic melanoma B

*
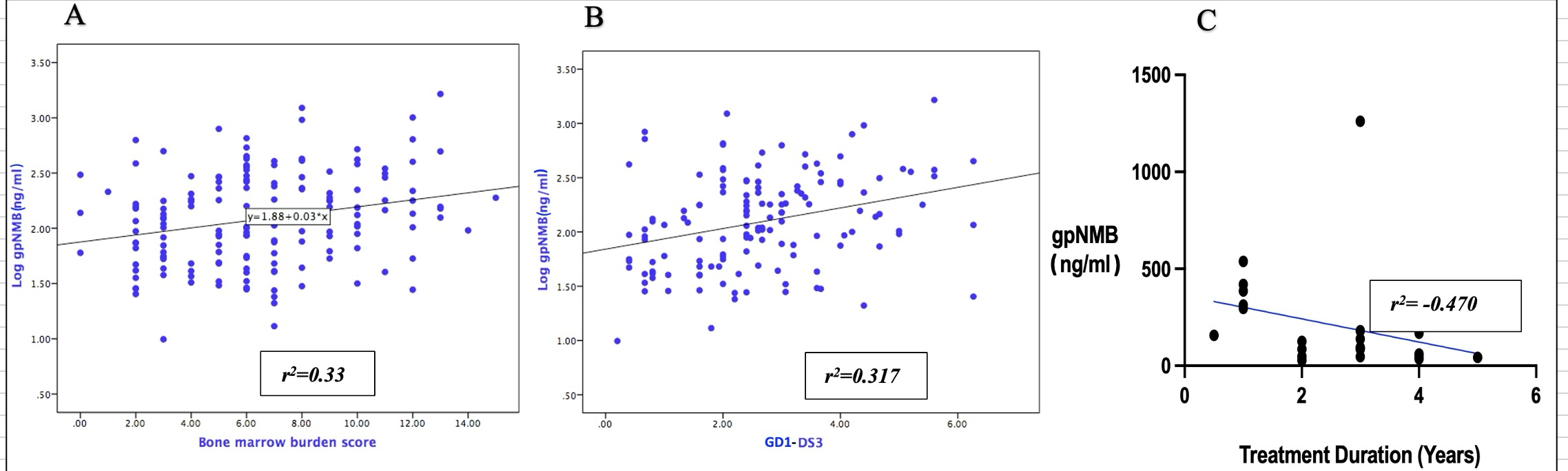
*

**Supplementary Fig. 2** Correlation of gpNMB with clinical parameters in Gaucher disease. (A) Bone marrow burden score correlation with gpNMB levels (B) Correlation of gpNMB Levels with Gaucher disease Type 1 severity scoring system (GD1-DS3), (C) Correlation of gpNMB Levels with treatment duration (≤ 5 years) *(rho=*-0.470*, p=*0.024*).* gpNMB: Glycoprotein nonmetastatic melanoma B, DS3: Severity Scoring System
